# Direct-to-consumer carrier screening for cystic fibrosis via a hospital website: a 6-year evaluation

**DOI:** 10.1007/s12687-018-0388-y

**Published:** 2018-09-18

**Authors:** Kim C. A. Holtkamp, Lidewij Henneman, Johan J. P. Gille, Hanne Meijers-Heijboer, Martina C. Cornel, Phillis Lakeman

**Affiliations:** 1 Department of Clinical Genetics, VU University Medical Center, PO Box 7057, 1007 MB, Amsterdam, The Netherlands; 2 Amsterdam Public Health Research Institute, Amsterdam, The Netherlands; 3 Amsterdam Reproduction and Development, Amsterdam, The Netherlands; 4 Department of Clinical Genetics, Academic Medical Center, Amsterdam, The Netherlands

**Keywords:** Carrier screening, Cystic fibrosis, Direct-to-consumer, Website, Evaluation, Preconception

## Abstract

A Dutch university hospital started offering cystic fibrosis (CF) carrier screening directly to consumers (DTC) through their website in 2010. A 6-year process evaluation was conducted to evaluate the offer. Screening was implemented as intended. However, uptake was lower than expected. Forty-four tests have been requested, partly by couples with a positive family history for CF, which was not the intended target group. Users were generally positive about the screening offer, citing accessibility, ease of testing, anonymity, and perceived shortcomings of regular healthcare as reasons for requesting screening. DTC CF carrier screening via a university hospital website is feasible, but is seldom used. Considering technological advances, continuation of this specific offer is questionable.

**Electronic supplementary material:**

The online version of this article (10.1007/s12687-018-0388-y) contains supplementary material, which is available to authorized users.

## Introduction

Cystic fibrosis (CF) is the most common autosomal recessive disorder among people of Northern European ancestry, with an overall carrier frequency of 1 in 25–30, and a birth prevalence of 1 in 2500–3600 (Massie and Delatycki 2013; Maxwell et al. 2011). Carrier screening aims to identify carrier couples who face a 1-in-4 risk of having affected offspring in each pregnancy, enabling informed reproductive decision-making. Preconception carrier screening allows the most complete range of reproductive options including prenatal diagnosis, preimplantation genetic diagnosis, the use of donor gametes, or refraining from having children.

Since the identification of the *CFTR* gene in the late 1980s (Riordan et al. 1989), the implementation of a population-based CF carrier screening has been widely discussed (Beaudet 1990; Massie and Delatycki 2013). Earlier studies showed that both the public (Henneman et al. 2003; Ioannou et al. 2014; Lakeman et al. 2009; Maxwell et al. 2011) and healthcare professionals (Janssens et al. 2014) have positive attitudes towards offering screening, either before pregnancy or in the early stages thereof. Most countries however, have not implemented CF carrier screening programs in regular healthcare, although there are exceptions. In the USA, CF carrier screening was introduced into routine care in 2001 (American College of Obstetricians and Gynecologists 2011). In Italy (Castellani et al. 2009) and Australia (Massie et al. 2014), it is offered regionally, and in Israel to all citizens (Zlotogora and Israeli 2009). In the Netherlands, despite several pilot studies (Henneman et al. 2003; Lakeman et al. 2008) and a recommendation from the Health Council (Health Council of the Netherlands 2007) to conduct a large-scale study on CF carrier screening, no population-based offer exists.

In recent years, direct-to-consumer (DTC) genetic testing, including carrier screening for CF, has become commercially available outside the traditional healthcare setting through the internet (Borry et al. 2011) and without the need for a licensed health professional (Borry et al. 2010). DTC testing has been criticized by both professional and public bodies (Rafiq et al. 2015; Skirton et al. 2012). Concerns have been expressed about the pre- and post-test information and counseling; medical supervision; consumer’s privacy and confidentiality; the test’s quality; and the possible impact on regular healthcare (Borry et al. 2011; Skirton et al. 2012).

In the Netherlands, in regular healthcare, carrier screening has only been available for family members and partners of *CFTR* mutation carriers and CF patients. In response to DTC companies, the Clinical Genetics Department of VU University Medical Center (VUMC) in Amsterdam started offering preconception CF carrier screening directly to consumers through their hospital website in 2010. Testing was aimed at couples planning a pregnancy without a positive family history for CF. The objective of this study is to evaluate the implementation of this DTC CF carrier screening offer.

## Methods

### The DTC CF carrier screening offer

Since December 2010, Dutch couples without a positive family history planning a pregnancy have been able to request CF carrier screening via the VUMC website (www.vumc.nl/CFtest [in Dutch]). The offer was developed by clinical and molecular geneticists, a health scientist, and a psychologist without commercial goals, and with the availability of pre-test counseling or contacting a clinical geneticist by e-mail or phone upon request. The offer was accompanied by online medical information compiled in accordance with the European guidelines (Castellani et al. 2010), and the costs are 150 euros per couple. A schematic representation is presented in Supplementary Table S1. Individuals with a positive family history of CF who contacted the patient coordinator for more information were referred to regular genetic counseling at the Clinical Genetic Department instead of testing via the website. They were explicitly told that it is important to know the exact *CFTR* mutations of their family members before testing, and that our standard screening test of 35 most frequent *CFTR* mutations in the Netherlands might not include their family members’ mutation.

At-home buccal swap sampling kits were used for the collection of both partners’ DNA samples. The procedure was to first test the woman by means of INNO-LiPA CFTR (Innogenetics, INNO-LiPA) which contains 35 *CFTR* mutations, including the most common mutations in the Netherlands, with an estimated sensitivity of 96%. A list of mutations and information on the test’s sensitivity, including information for non-Caucasian individuals (for whom the sensitivity is lower), was provided on the website. The DNA of the male partner was only tested by means of INNO-LIPA CFTR (without extra costs) when a *CFTR* mutation was identified with this test in the woman. In addition, a sex determination PCR test was performed to exclude sample swap of the male and female samples. After approximately 6 weeks, results were provided by phone by the clinical geneticist. Additionally, a letter was sent by e-mail, including information about a residual carrier risk with a negative result. In the case of an identified carrier couple, post-test counseling was offered at the Clinical Genetics Department.

### Study design

The DTC CF carrier screening offer was evaluated in terms of six implementation process indicators: (a) Fidelity (i.e., to what extent the offer was implemented as intended); (b) dose delivered (i.e., the number of test requests); (c) dose received (i.e., experiences of users); (d) reach (i.e., the degree to which the intended target group was reached); (e) recruitment (i.e., procedures used for recruitment); and (f) context (i.e., what contextual factors influenced implementation) (Hulscher et al. 2003; Saunders et al. 2005). These process indicators were translated into key evaluation questions to perform a 6-year evaluation. Both quantitative and qualitative research methodologies were used (see Table 1). The VU University Medical Center Medical Ethical Committee approved the study protocol.

**Table 1:** Process evaluation indicators, research questions, and data sources used to evaluate the DTC CF carrier screening offer

	Data sources (measures)				
	Uptake records (Document analysis)	Website pages (Document analysis)	Questions received via email website (Document analysis)	Key implementers (Semi-structured interviews)	Users (Semi-structured interviews)
Fidelity					
To what extent was the online CF carrier screening offer implemented as intended/ planned?				X	
Number of requests (dose delivered)					
How many CF carrier screening tests have been requested?	X				
How many people have been refused to have testing via the online offer?	X		X	X	
How many CF carrier screening tests have been performed?	X				
What were barriers for the execution of the CF carrier screening test?				X	
Experiences of users (dose received)					
How is an online offer of CF carrier screening perceived?				X	X
What were reasons to request/accept testing?			X		X
What needs do attendees have regarding information and counselling?					X
Reach					
To what extent is the target group - couples planning a pregnancy without positive family history - reached by the online offer?	X				X
How many people were reached by the information about the online offer?		X	X		X
Recruitment					
How are people informed about/ recruited for the online offer		X			X
Context					
What factors of the physical, social, and political environment, either directly or indirectly, affected the implementation?				X	X

### Data collection, preparation and analysis

The data sources to evaluate the key evaluation questions were: (1) uptake records; (2) website pages; (3) questions e-mailed by website visitors; and (4) semi-structured interviews among key implementers and test users (see Table 1).

The uptake records were analyzed to identify how many CF carrier screening tests were requested, approved, and performed. These records comprise data regarding the number of requested and performed screening tests, year of request, and the test results.

Website data, i.e., the number of (unique) page views, and time spent per page were obtained from OneStat and Google Analytics. In total, 51 e-mailed questions were received via the website from 39 individuals (potential users of the test (*n* = 36), students (*n* = 2), and a professional (*n* = 1)) between December 2010 and December 2016. All questions were assigned an identification number (CFx). Data was content analyzed, and coded by two researchers independently (LH/KH). Discrepancies in coding were discussed until consensus was reached.

Two key implementers (the laboratory specialist (JG) and clinical geneticist (PL) who could be consulted) were interviewed about their role in the process, the pre-set goals, and the perceived enabling and constraining factors in the implementation. Semi-structured interviews with test users (20–60 min) were conducted by a single researcher (KH/WO) to evaluate their experiences. In total, 24 out of 44 couples (55%) gave permission on the consent form to be contacted for evaluation research and were contacted by e-mail or post. Fourteen individuals responded, of whom 13 were willing to participate. The interviews were audio recorded and typed-out verbatim. Table 2 shows interviewees’ sociodemographic characteristics. A thematic content analysis was performed using the qualitative software program ATLAS.ti version 7.5 for Windows. All interviews were coded by two researchers independently (LH/KH).

**Table 2:** Characteristics of interviewed users (*n* = 13)

	***n***
Gender^1^	
Male	2
Female	11
Age (years)	
26–35	8
≥ 36	5
Education^2^	
Low/intermediate	1
High	9
Missing	3
Religious activity	
Very/somewhat active	1
Not active/not applicable	9
Missing	3
Marital status	
Cohabiting/married	12
Divorced	1
Having children	
Yes	9
No	2
Missing	2
Positive family history for CF	
Yes	7
No	6

^1^One male and one female are a couple but interviewed separately

^2^Low: primary school, lower level of secondary school, lower vocational training. Intermediate: higher level of secondary school, intermediate vocational training. High: higher vocational training, university

## Results

The results are presented according to the six process indicators.

### Fidelity

The goals of the DTC CF carrier screening offer were: (1) realization of an CF carrier screening offer for couples planning to have children without a positive family history for CF; (2) gaining experience with offering CF carrier screening via a website and by means of at-home buccal swap kits; and (3) the provision of carrier screening with sufficient counseling and follow-up in reaction to the upcoming commercial offers. Considering these goals, it can be concluded that all have been achieved. As one of the key implementers (KI) stated: “We have shown that it is possible to offer carrier screening outside the regular healthcare setting, without providing face-to-face pre-test counselling” (KI 1).

### Number of test requests (dose delivered)

In a period of 6 years, 44 CF carrier tests were requested (between three and ten per year) by 39 couples, and five individuals (due to donor gamete procedures) (Supplementary Fig. S1). Three requests were declined due to pregnancy (*n* = 1) or positive CF family history (*n* = 2). Forty-one requests were approved, and buccal samples were collected. Mean age for men and women was 36 (SD = 7.9) and 32 (SD = 7.3) years, respectively. In total, 65 tests were performed. In 12 cases, the initial analyses failed due to bad quality of the buccal sample. The same samples were either tested again or the partner’s sample was tested instead. In one case, the analysis of both the male and female sample failed and new buccal samples were requested. In another case, the analysis of the female sample failed twice, but was tested for a third time because her partner was identified as a CF carrier. Finally, in one case the couple had switched their samples, and thus both samples were tested. Five carriers, all of the F508del mutation, were identified (Supplementary Fig. S1). No carrier couples were found.

Website visitors’ questions are shown in Table 3. The topic most often addressed was whether it was possible to request the online CF carrier screening test in the case of a positive family history (*n* = 22, 43%), of which five were also about testing in pregnancy.

**Table 3:** Topics that website visitors (*n* = 39) inquire about

Topics	(total *n* = 51)	
Is it possible to request DTC CF carrier testing:	27	
- If I have a positive family history for CF/my partner is a carrier?		17
- If I or my partner is pregnant and with positive family history/my partner is CF carrier?		5
- For only one individual?^1^		2
- If I do not live in the Netherlands?		2
- If I or my partner is pregnant?		21
Costs and reimbursement of testing	3	
Aspects of testing:	7	
- Procedures regarding testing^2^		3
- Are two samples needed in the case of individual testing?		2
- What is the turnaround time?		2
Other testing possibilities:	4	
- What tests can be performed on a fetus?		2
- Carrier screening for other disorders possible?		1
- Availability of carrier screening elsewhere in the Netherlands?		1
Practical requests:	4	
- Is referral from a general practitioner necessary?		1
- Can you write a “family letter”?		1
- When do I receive the at-home buccal swap sampling kit?		1
- Can I get insight into the privacy regulations?		1
Other^3^	3	

^1^Instead of couple testing

^2^E.g., questions about the process of testing, the order of testing (female and male saliva sample), and the sending of samples (through post or personal delivery)

^3^Information on needs of CF patients and inheritance of CF

### Experiences with the online DTC CF carrier screening offer (dose received)

The two key implementers were positive about the offer. However, reflecting on the uptake, one stated “It would not be an acute problem if we shut down this offer” (KI 1). The other added that it would make more sense to embed it in, for example, an expanded offer of carrier screening where multiple disorders are screened simultaneously. The financial sustainability of offering CF carrier screening in the current way was questioned as laboratory costs are higher than the revenues.

The test users perceived the offer as positive as the availability of this offer enables people to decide for themselves whether to request CF carrier screening or not (Table 4, quote 1). While one woman was surprised that only few tests had been requested (Table 4, quote 2), another wondered whether there actually was a demand for screening, and thus if an offer outside regular healthcare should be realized (Table 4, quote 3). From the interviews, it appeared that users were often familiar with CF and carrier screening, either due to a positive family history for CF or experiences via work or studies. Many also argued that these experiences had influenced their carrier screening request (Table 4, quote 3). Although the DTC offer was aimed at couples without a positive family history, half of the interviewed users turned out to have a positive family history (see Table 2). Reasons for requesting the online CF offer included the accessibility and ease of the test, a feeling of anonymity, and shortcomings of regular healthcare (Table 4, quotes 4–9). Regarding the latter, users felt that waiting lists in regular healthcare were too long, costs were too high, or perceived the process of obtaining a referral from the general practitioner as a barrier for regular screening (Table 4, quotes 6, 7). If this process were to be improved, regular screening would have been preferred over the online offer by some users since this is more common. Furthermore, due to a lack of knowledge among healthcare professionals about CF carrier screening, some users felt compelled to search for testing possibilities outside regular healthcare, an observation that also emerged from the website questions (Table 3).

**Table 4:** Themes and illustrative quotes of user interviews (*n* = 13)

Theme	No.	Illustrative quotes
Availability of the DTC CF carrier screening offer		
	1	“That people at least have the possibility to get informed and to request testing when they want to, to make sure that they are able to make an informed decision. […] It just matches the trends of this time.” (#8, woman, − FamHis CF, not carrier)
	2	“I was surprised that only about 50 couples had requested testing via the website. I was really surprised that it was this low. It’s non-invasive, and not time-consuming, but I think it might be caused by unawareness” (#12, woman, − FamHis CF, not carrier)
	3	“I was just surprised about the availability of the offer, and the ease with which one can test. I thought, if we all do this, what do we do with the information in the end? […] What more do we want to test then? […] I thought is there such a demand that it can be this easily investigated? […] If she [niece] did not have CF, I would not have wanted to know this out of my own curiosity” (#10, woman, + FamHis CF, not carrier)
Reasons to request DTC CF carrier screening:		
Easy to request	4	“I found it really nice. In hindsight, it was ideal, because you did not have to go to the hospital. It [the buccal sampling kit] is sent via post, it actually is really easy.” (#9, woman, + FamHis CF women, not carrier)
Feeling of anonymity	5	“It was easy to request, that was nice but it was also fairly anonymous. You did not have to go the general practitioner.” (#9, woman, − FamHis CF, not carrier)
Perceived shortcomings of regular healthcare:		
Waiting lists are too long	6	“We requested testing via the Genetics Department but they had a very long waiting list. The pediatrician then said, try this [DTC CF carrier screening] first, you will have the results sooner.” (#1, women, − FamHis CF, not carrier).
Too expensive	7	“I had an appointment [at the Genetics Department] but it turned out that if I were to request testing via that way, it would cost a lot of money. […] I believe that the insurance would reimburse it, but I had a maximum policy excess, so it would cost 800 euros or so.” (#9, women, + FamHis CF, not carrier)
No target group	8	“We did go to the general practitioner, but she said: “Such a test [carrier test] is only performed for couples planning a pregnancy” [partners were 64 and 65 years old], so she could not help us […] and so we ended up with you.” (#6, women, + FamHis CF, not carrier)
Lack of knowledge among professionals	9	“We noticed that it [carrier testing] was quite new for her [general practitioner], and that she did not know how to handle the situation.” (#13, man, + FamHis CF, not carrier)
Information and counseling:		
Clear and accessible information on the website	10	“The website is very accessible. As I remember, it was written in simple language. That was really nice.” (#5, woman, + FamHis CF, not carrier)
Need for clear contact details patient coordinator	11	“I contacted the clinical geneticist because I did not know whom to contact. […] But I do remember that it wasn’t clearly mentioned on the website who to contact in the case of questions.” (#10, woman + FamHis CF, not carrier)
Need for personal contact for clarifying questions	12	“As we experienced it, sometimes you are close to a situation described in the information on the website […] that you would like to have personal contact either via telephone or face-to-face. Especially with these kinds of issues.” (#13, man, + FamHis CF, not carrier)
Need for more information about exclusion of couples with a positive family history for CF	13	“When you do have someone with CF in your family, I do not understand why you cannot have this test as well. I do not see the added value of going to the clinical geneticist, which costs a lot of money, while I think: if you have someone in your family, you know enough, and of course this is just as good a choice.” (#10, woman, + FamHis CF, not carrier)
Need for more information about procedure of testing	14	“I expected that we would both get a result, so I was a bit surprised that we were told: ‘only you get the results, and if you are not identified as a carrier, we do not test your husband’. It does make sense, but that caught me by surprise.” (#7, woman, − FamHis CF, not carrier)
Need for information regarding carrier screening for other disorders	15	“I would like to have had more information on other possibilities of carrier screening, I could have looked at those as well then.” (#12, woman, − FamHis CF, not carrier)

Users were positive about the information on the website, and described it as clear, understandable, realistic, approachable, and sufficient. However, some mentioned that the contact details of the patient coordinator were difficult to find (Table 4, quote 11). Furthermore, some would have liked more information on why people with a positive family history were excluded (Table 4, quote 13), and on testing possibilities for other disorders (Table 4, quote 15). Finally, it appeared that the information on the sequential procedure of testing was not clear to everyone (Table 4, quote 14).

### Reach and recruitment

In total, 9410 page views lasting 1.24 min (range 19 s–3 mins) per page were measured on average per year. The key implementers had expected far more people to order the test, based on positive attitudes in earlier studies (Henneman et al. 2003; Lakeman et al. 2009), and found it difficult to determine whether the target group has been reached as only 44 tests were requested. Additionally, a proportion of these people do not belong to the initial target group due to having a positive family history. Users mostly found the offer via an Internet search but some were informed by their healthcare providers (e.g., general practitioner, clinical geneticist, or pediatrician).

### Context

According to the key implementers, a number of contextual factors affected the implementation of the online CF offer. An offer of CF carrier screening by means of buccal swap samples was achievable. However, as one of the key implementers explained, extra attention should be paid to the DNA isolation process, as normally blood samples are used and slightly different procedures need to be followed by technicians. Furthermore, to increase and maintain awareness regarding the online CF offer, a media plan had been developed. However, a lack of (skilled) staff and time resulted in its limited use. The availability of the website, the announcement in some (local) newspapers, and informing general practitioners and midwives in educational sessions was not enough to increase awareness. Moreover, as there were no commercial goals linked to this offer, and mixed messages had to be avoided, the implementers found it difficult to advertise and actively seek publicity. Initially, it was planned to embed the information about the existence of DTC CF carrier screening in preconception care consultations offered by midwives and general practitioners in the Netherlands. However, these consultations were visited by few people considering a pregnancy and few were thus informed about the offer.

## Discussion

A 6-year evaluation of the DTC CF carrier screening offer via a Dutch university hospital website showed that it is feasible to develop and offer screening by means of at-home buccal sampling kits, although very few tests were requested. Interviews showed that half of the users had a positive family history for CF, while screening was aimed at couples without a positive family history. Users were generally positive about the offer, but also cited shortcomings of regular healthcare as a motive to seek DTC testing (e.g., long waiting lists, high costs of testing, and lack of knowledge among professionals).

Analysis of the performed tests showed that 15 out of 65 tests failed in the first instance because insufficient DNA could be recovered from the buccal samples or samples had been swapped. The preparation of couples (e.g., eating/drinking before taking a test, rinsing of the mouth) and/or not correctly following the sample swap instructions may have resulted in the insufficient samples, but we have no proof for these assumptions. Collection of buccal swabs by professionals (e.g., general practitioners or nurses) might increase its success rate, although this may limit the accessibility of screening. Another option, using blood samples, might raise a barrier to request screening (Clayton et al. 1996). The turnaround time for carrier test results could be shorter than 6 weeks if a sufficient number of samples is sent to make this cost effective. Despite the positive public attitudes reported in other studies (Henneman et al. 2003; Lakeman et al. 2009; Maxwell et al. 2011), only 44 couples/individuals requested CF carrier screening in 6 years. McClaren et al. (2008) showed that potential consumers perceived carrier screening as “not in my world,” and were unlikely to request testing, unless offered by a healthcare professional or if they had a positive family history. Moreover, a study among users of DTC personal genomic testing showed that only one third of the responders was interested in obtaining carrier status information (Roberts et al. 2017). The authors argue that this information may not be viewed as personally relevant, or users are unfamiliar with these tests compared to other types of genetic tests (e.g., tests to determine disease risks) (Roberts et al. 2017). Additionally, we have previously shown that familiarity with genetic disorders and screening may be a critical factor in the implementation of carrier screening (Holtkamp et al. 2017). The lack of familiarity with CF or carrier screening might thus have impeded the implementation of the DTC CF carrier screening offer.

In comparison to visitor numbers on a website regarding non-invasive prenatal testing (NIPT), with about 1000 visitors per month (Tamminga et al. 2017), the website on DTC CF carrier screening has reached fewer people. Though a media plan had been developed, and actions were undertaken to increase awareness about the offer (e.g., via newspaper and magazine articles), this media plan was not fully executed. As argued by one of the key implementers, in hindsight, extra personnel or even a marketing company is needed to do so. The use of a marketing company, however, might conflict with the non-commercial goals of the offer. To what extent do you actively inform people? Attention should be paid to maintain its basic (ethical) principles (e.g., autonomy and informed choice).

A relatively large number of users had a positive family history for CF and used the offer as a form of cascade testing. Especially among these users, shortcomings of regular healthcare were mentioned regularly as the primary reason for requesting the DTC offer. This raises the question of whether regular healthcare should adapt its possibilities for carrier screening. One might consider a separate, fast route for carrier screening of family members.

During the 6-year period that the DTC CF test was available, technological advances have resulted in the development of expanded carrier screening panels allowing the detection of a much larger set of sequence variants and multiple disorders simultaneously at a faster turnaround time and without significantly increasing costs (Henneman et al. 2016). As the financial sustainability of the current DTC CF offer was questioned, one might consider integrating CF in these expanded panels, and terminate the separate CF. In the meantime, Amsterdam University Medical Centers have started to offer carrier screening for 50 disorders, with pre-test genetic counseling offered by clinical geneticists. Research has shown that one third of the respondents in a Dutch survey study on how potential users from the general population view a test for 50 severe, early lethal diseases, would hypothetically take an expanded carrier screening test if it were to be offered (Plantinga et al. 2016). Further research will need to show the actual interest and uptake of such an expanded screening offer.

By performing a process evaluation of the DTC CF carrier screening offer, a detailed description of its implementation could be provided. The use of both quantitative and qualitative methods enabled the in-depth study of the offer and strengthens the conclusions. Because it is a case study, the sample sizes are small, and it concerns a specific setting in one hospital. These data should thus not be used for generalization.

In conclusion, this evaluation of a DTC CF carrier screening offer via a hospital website has shown its feasibility. The low uptake, and the fact that the offer is not primarily used by people without a positive family history of CF, however, raise questions regarding its future existence in this particular format. Ongoing technological advances enable the inclusion of CF in expanded universal carrier screening panels.

## Electronic supplementary material

Supplementary Table S1 (DOCX 30 kb)

Supplementary Figure S1 (DOCX 284 kb)

## References

[CR1] American College of Obstetricians and Gynecologists (2011). Committee opinion no. 486. Update on carrier screening for cystic fibrosis. Obstet Gynecol.

[CR2] Beaudet AL (1990). Invited editorial: carrier screening for cystic fibrosis. Am J Hum Genet.

[CR3] Borry P, Cornel MC, Howard HC (2010). Where are you going, where have you been: a recent history of the direct-to-consumer genetic testing market. J Community Genet.

[CR4] Borry P, Henneman L, Lakeman P, ten Kate LP, Cornel MC, Howard HC (2011). Preconceptional genetic carrier testing and the commercial offer directly-to-consumers. Hum Reprod.

[CR5] Castellani C, Picci L, Tamanini A, Girardi P, Rizzotti P, Assael BM (2009). Association between carrier screening and incidence of cystic fibrosis. JAMA.

[CR6] Castellani C, Macek M, Cassiman JJ, Duff A, Massie J, ten Kate LP, Barton D, Cutting G, Dallapiccola B, Dequeker E, Girodon E, Grody W, Highsmith EW, Kaariainen H, Kruip S, Morris M, Pignatti PF, Pypops U, Schwarz M, Soller M, Stuhrman M, Cuppens H (2010). Benchmarks for cystic fibrosis carrier screening: a European consensus document. J Cyst Fibros.

[CR7] Clayton EW, Hannig VL, Pfotenhauer JP, Parker RA, Campbell PW, Phillips JA (1996). Lack of interest by nonpregnant couples in population-based cystic fibrosis carrier screening. Am J Hum Genet.

[CR8] Health Council of the Netherlands (2007) Preconception care: a good beginning. The Haque: Publication no 2007/19

[CR9] Henneman L, Bramsen I, van KL, van Acker MB, Pals G, van der Horst HE, Ader HJ, van der Ploeg HM, ten Kate LP (2003). Offering preconceptional cystic fibrosis carrier couple screening in the absence of established preconceptional care services. Community Genet.

[CR10] Henneman L, Borry P, Chokoshvili D, Cornel MC, van El CG, Forzano F, Hall A, Howard HC, Janssens S, Kayserili H, Lakeman P, Lucassen A, Metcalfe SA, Vidmar L, de Wert G, Dondorp WJ, Peterlin B (2016). Responsible implementation of expanded carrier screening. Eur J Hum Genet.

[CR11] Holtkamp KC, Mathijssen IB, Lakeman P, van Maarle MC, Dondorp WJ, Henneman L, Cornel MC (2017). Factors for successful implementation of population-based expanded carrier screening: learning from existing initiatives. Eur J Pub Health.

[CR12] Hulscher M, Laurant M, Grol R (2003). Process evaluation on quality improvement interventions. Qual Saf Health Care.

[CR13] Ioannou L, McClaren BJ, Massie J, Lewis S, Metcalfe SA, Forrest L, Delatycki MB (2014). Population-based carrier screening for cystic fibrosis: a systematic review of 23 years of research. Genet Med.

[CR14] Janssens S, De Paepe A, Borry P (2014). Attitudes of health care professionals toward carrier screening for cystic fibrosis. A review of the literature. J. Community Genet.

[CR15] Lakeman P, Plass AM, Henneman L, Bezemer PD, Cornel MC, ten Kate LP (2008). Three-month follow-up of Western and non-Western participants in a study on preconceptional ancestry-based carrier couple screening for cystic fibrosis and hemoglobinopathies in the Netherlands. Genet Med.

[CR16] Lakeman P, Plass AM, Henneman L, Bezemer PD, Cornel MC, ten Kate LP (2009). Preconceptional ancestry-based carrier couple screening for cystic fibrosis and haemoglobinopathies: what determines the intention to participate or not and actual participation?. Eur J Hum Genet.

[CR17] Massie J, Delatycki MB (2013). Cystic fibrosis carrier screening. Paediatr Respir Rev.

[CR18] Massie J, Ioannou L, Delatycki M (2014). Prenatal and preconception population carrier screening for cystic fibrosis in Australia: where are we up to?. Aust N Z J Obstet Gynaecol.

[CR19] Maxwell SJ, Kyne G, Molster C, Barker NM, Ormsby J, O'Leary P (2011). Perceptions of population cystic fibrosis prenatal and preconception carrier screening among individuals with cystic fibrosis and their family members. Genet Test Mol Biomarkers.

[CR20] McClaren BJ, Delatycki MB, Collins V, Metcalfe SA, Aitken M (2008). "It is not in my world": an exploration of attitudes and influences associated with cystic fibrosis carrier screening. Eur J Hum Genet.

[CR21] Plantinga M, Birnie E, Abbott KM, Sinke RJ, Lucassen AM, Schuurmans J, Kaplan S, Verkerk MA, Ranchor AV, van Langen IM (2016). Population-based preconception carrier screening: how potential users from the general population view a test for 50 serious diseases. Eur J Hum Genet.

[CR22] Rafiq M, Ianuale C, Ricciardi W, Boccia S (2015). Direct-to-consumer genetic testing: a sytematic review of European guidelines, recommendations, and position statements. Genet Test Mol Biomarkers.

[CR23] Riordan JR, Rommens JM, Kerem B, Alon N, Rozmahel R, Grzelczak Z, Zielenski J, Lok S, Plavsic N, Chou JL (1989). Identification of the cystic fibrosis gene: cloning and characterization of complementary DNA. Science.

[CR24] Roberts JS, Gornick MC, Carere DA, Uhlmann WR, Ruffin MT, Green RC (2017). Direct-to-consumer genetic testing: user motivations, decision making, and perceived utility of results. Public Health Genomics.

[CR25] Saunders RP, Evans MH, Joshi P (2005). Developing a process-evaluation plan for assessing health promotion program implementation: a how-to guide. Health Promot Pract.

[CR26] Skirton H, Goldsmith L, Jackson L, O'Connor A (2012). Direct to consumer genetic testing: a systematic review of position statements, policies and recommendations. Clin Genet.

[CR27] Tamminga S, van Dussen L, Verweij EJ, de Boer MA, Cornel MC, Henneman L, For the Dutch NC (2017). What do people want to know about NIPT? Content analysis of questions emailed to national NIPT information websites. Prenat Diagn.

[CR28] Zlotogora J, Israeli A (2009). A comprehensive screening program for cystic fibrosis. IMAJ.

