# Supplementary material for: Direct-to-consumer carrier screening for cystic fibrosis via a hospital website: a 6-year evaluation
Source: J Community Genet. 2018 Sep 18;10(2):249–57. doi: 10.1007/s12687-018-0388-y (PMC6435772; doi:10.1007/s12687-018-0388-y)
Supplement: Supplementary file 1 — (DOCX 30 kb) [file 12687_2018_388_MOESM1_ESM.docx]

**Supplementary Table S1**.

Manuscript: **Direct-to-consumer carrier screening for cystic fibrosis via a hospital website: a six-year evaluation**

**Journal of Community Genetics**

Kim C.A. Holtkamp^1,2,3^, Lidewij Henneman^1,2,3^, Johan J.P. Gille^1^, Hanne Meijers-Heijboer^1,4^, Martina C. Cornel^1,2^, Phillis Lakeman^3,4^

^1^Department of Clinical Genetics, VU University Medical Center, Amsterdam, The Netherlands

^2^Amsterdam Public Health research institute, Amsterdam, The Netherlands

^3^Amsterdam Reproduction and Development, Amsterdam, The Netherlands

^4^Department of Clinical Genetics, Academic Medical Center, Amsterdam, The Netherlands

**Corresponding author:** Email: l.henneman@vumc.nl

Supplementary Table S1. Schematic representation of the DTC CF carrier screening offer^a^

| Problem | Evidence base | Input | Output  Activities | 0  Participation | Intended outcomes  Short-term | Mid-/long-term |
| --- | --- | --- | --- | --- | --- | --- |
| *What is the problem?* | *Scientific evidence* | *What is invested?* | *What is done?* | *Who is reached?* | *What are the short-term intended outcomes?* | *What are the mid-/long-term intended outcomes?* |
| Prior to 2010, CF carrier screening was not available for couples planning a pregnancy without a positive family history for CF  Children with CF are born unexpectedly  Commercial companies have been offering direct-to-consumer (DTC) genetic testing (including testing for CF), but have been criticised by professionals | About 1 in 900 couples of North-European descent have a 1-in-4 risk of having a child with CF each pregnancy  Carrier screening allows couples to find out whether they are a carrier couple and thus at increased risk of having an affected child each pregnancy | Staff  Time  Money  Equipment  Materials  Technology | Providing medical information via a university hospital website  Offering an at-home buccal swap sampling kit for CF carrier screening via a university hospital website for 150 euros per couple  Testing for 35 most common mutations in the *CFTR* gene (the woman was tested first, the man was tested only when the woman was identified as a carrier).  Reporting test results directly to couples including information on residual risk  Providing counselling if necessary | Couples planning a pregnancy, without a positive family history for CF, who are interested in having CF carrier screening | Increase of couples’ knowledge about CF carrier status, and the possible increased risk of having a child with CF  Possibility of cascade screening among family members of identified CF carriers  Experiences among professionals with offering carrier screening via a university hospital website | Facilitating informed reproductive decision-making for couples planning a pregnancy  Reducing the number of children that are unexpectedly born with CF |

^a^ Schematic representation based on a Logic Model which is a systematic and visual way to present the relationships among the input used to operate a programme or project, the activities planned and the changes or results (intended outcomes) that will be achieved (Kellogg Foundation WK 2004).
