# Supplementary material for: Direct-to-consumer carrier screening for cystic fibrosis via a hospital website: a 6-year evaluation
Source: J Community Genet. 2018 Sep 18;10(2):249–57. doi: 10.1007/s12687-018-0388-y (PMC6435772; doi:10.1007/s12687-018-0388-y)
Supplement: Supplementary file 2 — (DOCX 284 kb) [file 12687_2018_388_MOESM2_ESM.docx]

Supplementary Figure S1

Manuscript: **Direct-to-consumer carrier screening for cystic fibrosis via a hospital website: a six-year evaluation**

**Journal of Community Genetics**

Kim C.A. Holtkamp^1,2,3^, Lidewij Henneman^1,2,3^, Johan J.P. Gille^1^, Hanne Meijers-Heijboer^1,4^, Martina C. Cornel^1,2^, Phillis Lakeman^3,4^

^1^Department of Clinical Genetics, VU University Medical Center, Amsterdam, The Netherlands

^2^Amsterdam Public Health research institute, Amsterdam, The Netherlands

^3^Amsterdam Reproduction and Development, Amsterdam, The Netherlands

^4^Department of Clinical Genetics, Academic Medical Center, Amsterdam, The Netherlands

**Corresponding author:** Email: l.henneman@vumc.nl


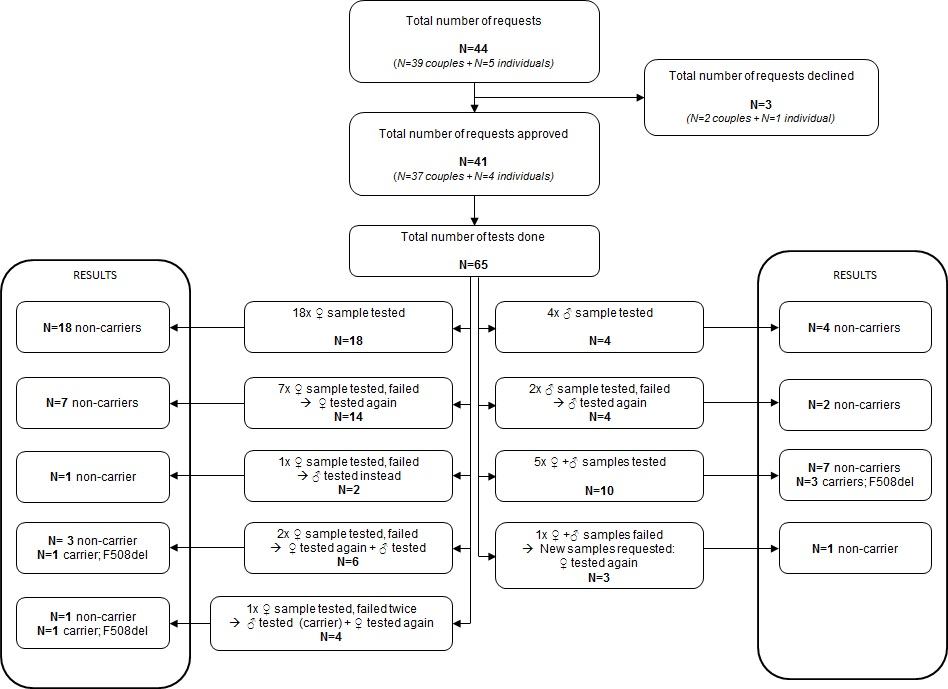


**Supplementary Figure S1**

Flow chart of requests and analyses of samples for cystic fibrosis carrier screening via the hospital website
